# Evaluating LiDAR Perception Algorithms for All-Weather Autonomy

**DOI:** 10.3390/s25247436

**Published:** 2025-12-06

**Authors:** Himanshu Gupta, Achim J. Lilienthal, Henrik Andreasson

**Affiliations:** 1 Centre for Applied Autonomous Sensor Systems, Örebro University, 70182 Örebro, Sweden; achim.lilienthal@oru.se (A.J.L.); henrik.andreasson@oru.se (H.A.); 2 Chair Perception for Intelligent Systems (PercInS), Munich Institute of Robotics and Machine Intelligence (MIRMI), Technical University of Munich, 80992 München, Germany

**Keywords:** 3D object detection, adverse weather, LiDAR perception, localization, point cloud filter, SLAM

## Abstract

LiDAR is used in autonomous driving for navigation, obstacle avoidance, and environment mapping. However, adverse weather conditions introduce noise into sensor data, potentially degrading the performance of perception algorithms and compromising the safety and reliability of autonomous driving systems. Hence, in this paper, we investigate the limitations of LiDAR perception algorithms in adverse weather conditions, explore ways to mitigate the effects of noise, and propose future research directions to achieve all-weather autonomy with LiDAR sensors. Using real-world datasets and synthetically generated dense fog, we characterize the noise in adverse weather such as snow, rain, and fog; their effect on sensor data; and how to effectively mitigate the noise for tasks like object detection, localization, and SLAM. Specifically, we investigate point cloud filtering methods and compare them based on their ability to denoise point clouds, focusing on processing time, accuracy, and limitations. Additionally, we evaluate the impact of adverse weather on state-of-the-art 3D object detection, localization, and SLAM methods, as well as the effect of point cloud filtering on the algorithms’ performance. We find that point cloud filtering methods are partially successful at removing noise due to adverse weather, but must be fine-tuned for the specific LiDAR, application scenario, and type of adverse weather. 3D object detection was negatively affected by adverse weather, but performance improved with dynamic filtering algorithms. We found that heavy snowfall does not affect localization when using a map constructed in clear weather, but it fails in dense fog due to a low number of feature points. SLAM also failed in thick fog outdoors, but it performed well in heavy snowfall. Filtering algorithms have varied effects on SLAM performance depending on the type of scan-matching algorithm.

## 1. Introduction

LiDAR is a crucial sensor for autonomous driving systems, providing 3D spatial information necessary for navigation, obstacle avoidance, and environment mapping. LiDAR sensors generate high-resolution point clouds of the surrounding environment. However, noise in sensor data caused by adverse weather conditions can degrade the performance of LiDAR perception algorithms, affecting the safety and effectiveness of autonomous driving systems.

**Figure 1:**
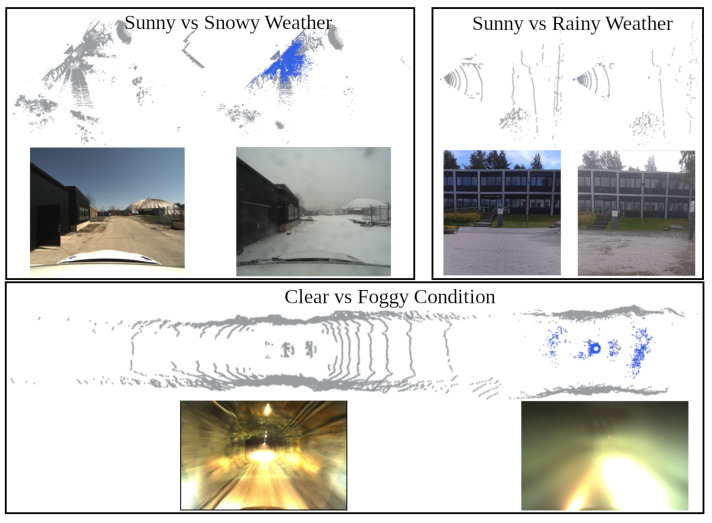
Point clouds and corresponding images in sunny vs. snowy (Boreas Dataset [4]), sunny vs. rainy (self-collected), and clear vs. foggy (self-collected in tunnel) conditions. The blue color shows noise due to adverse weather, which is minor even in heavy rain. The point cloud range in dense fog is reduced, resulting in a smaller point cloud.

Figure 1 shows the noise patterns in snowy, rainy, and foggy weather. Previous studies [1,2,3] have extensively investigated the effect of adverse weather on LiDAR sensors in lab-controlled environments for rain and fog. These studies provide insights into LiDAR’s usability in adverse weather, revealing that in very dense fog, LiDAR perception can be significantly compromised. The effective LiDAR range is reduced to 25 m when meteorological visibility is less than 40 m, as noted in [3] for Velodyne HDL64 LiDAR. While such conditions can reduce LiDAR’s operational range, LiDAR perception algorithms may not be significantly affected. Therefore, their limitations should be investigated for practical usability, making it the primary focus of this research.

In addition to studying the effect of adverse weather on LiDAR sensors, other studies focused on enhancing LiDAR perception under such conditions. For instance, Refs. [5,6] used both real and synthetic weather noise to train a LiDAR-based object detection model to improve performance in adverse weather conditions. However, the effect of point cloud filtering on object detection under adverse weather conditions was not investigated, which this work addresses. Additionally, we have tested the effectiveness of various filtering methods (statistical and deep learning) for their general applicability across different weather conditions and evaluated their limitations in downstream perception tasks.

In addition to evaluating point cloud filtering methods, we examined how adverse weather-induced noise affects LiDAR perception algorithms, such as object detection, localization, and SLAM. We assessed the effectiveness of point cloud filtering on algorithm performance and explored the limitations of LiDAR sensors in extreme weather conditions. For this purpose, we used real-world datasets collected under snowy, sunny, and rainy conditions, and synthetic data for foggy conditions.

In this work, we present our evaluation from an algorithmic perspective, making the following contributions to understanding the limitations of current LiDAR perception algorithms in adverse weather conditions using a public dataset collected with rotating automotive LiDARs operating at 905 nm.

1. We assess the impact of adverse weather and various filtering algorithms on the performance of state-of-the-art 3D object detection models, localization, and SLAM algorithms, offering insights into the robustness and limitations of LiDAR perception algorithms in various weather conditions.
2. We highlight the challenges of LiDAR perception in adverse weather and suggest practical solutions to mitigate their impact.
3. We re-implemented various point cloud filtering methods for faster execution and evaluated their effectiveness in mitigating noise caused by adverse weather conditions, specifically fog and snow. All code and configurations used in this study are available at https://github.com/hgupta01/LiDAR-in-Adverse-Weather-Evaluation, (accessed on 24 November 2025).

Section 2 presents the dataset used in this work. In Section 3, we analyzed the noise caused by adverse weather using real-world datasets. Section 4, Section 5, Section 6 and Section 7 present the evaluation of filtering, object detection, localization, and SLAM in adverse weather, respectively. Section 8 and Section 9 present an overall discussion of the experimental results and conclude the paper with future research directions to achieve all-weather autonomy using LiDAR sensors.

## 2. Datasets

In recent years, several LiDAR datasets have been released for tasks such as segmentation, object detection, and SLAM in adverse weather conditions. However, no dataset encompasses all weather conditions or weather severity, or supports all perception tasks. Therefore, we utilized multiple datasets to evaluate various LiDAR perception tasks as presented in Table 1.

To assess LiDAR perception algorithms in snowy weather, we utilized the Winter Adverse Driving Dataset (WADS) [8] to evaluate point cloud filters. The Canadian Adverse Driving Conditions (CADC) dataset [9] was used to assess object detection in snowy weather and to examine the impact of point cloud denoising on detection accuracy. The Boreas dataset [4] was used to evaluate localization and SLAM algorithms. The snowfall intensity in these datasets varies from moderate to heavy.

In contrast, only a few datasets include rainy conditions. The Radiate dataset [10] features low-to-moderate rain intensity. As discussed in Section 3, the noise pattern in rain is similar to that in snow. Hence, we did not evaluate filtering and object detection in rain, and we used the Boreas Dataset sequence “boreas-2021-04-29-15-55” to evaluate localization and SLAM algorithms.

**Figure 2:**
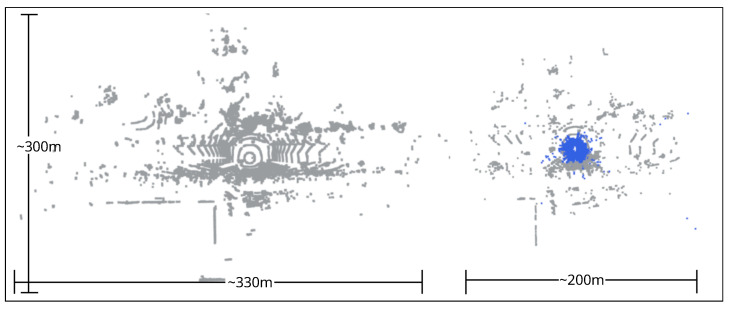
A top-view of a point cloud in Boreas dataset with and without simulated dense fog. Blue color represent the simulated fog noise.

Similarly, datasets with foggy conditions are limited. The Radiate dataset [10] contains low-intensity fog, while the Dense dataset [11] provides fog data from a controlled fog chamber, which is unsuitable for localization and SLAM and is also limited for object detection. Hence, we synthetically generated dense fog using a physics-based fog simulator, LiDAR-fog-sim [7]. We used the “boreas-objects-v1” and “boreas-2021-04-08-12-44” sequences of the Boreas dataset to evaluate filtering and object detection algorithms, as well as localization and SLAM. The ground truth for fog-induced noise is estimated using a nearest-neighbor approach between the original and simulated point clouds. The LiDAR-fog-sim does not generate new points, making it easy to get the ground truth. In object detection, we kept only bounding boxes with more than ten points. Figure 2 shows a point cloud with simulated fog.

Due to unavailability of data with dense fog and heavy rainfall in a real-world scenario, we have collected a short sequence of point cloud data with heavy rainfall and dense fog for adverse noise analysis and visualization used in Section 3. However, the self-collected dataset cannot be used for object detection and navigation studies.

## 3. Noise in Adverse Weather

Adverse weather affects LiDAR point cloud in three primary ways, as documented in previous literature [1,2,12,13]. The first and most severe effect is noisy points due to adverse weather that do not correspond to objects of interest, as illustrated in Figure 1. These noisy points, representing fog, dust, snowflakes, or raindrops, are transient and scattered.

The second effect is reduced LiDAR range in adverse weather, as shown in Figure 1, which visualizes point clouds in clear and adverse weather. The reduced point range affects LiDAR perception, including localization, SLAM, and object detection, which impacts the safety of autonomous driving systems. Previous studies [1,2,14] have demonstrated the relationship between point range and weather intensity in a controlled environment using a fog-chamber.

**Figure 3:**
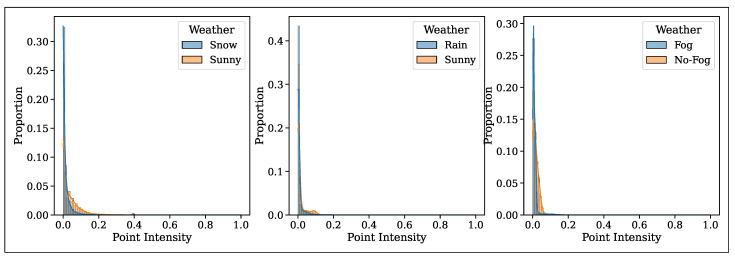
Normalized point intensity histogram in adverse weather and sunny/clear weather using the data shown in Figure 1.

Another negative effect of adverse weather is reduced point intensity as reported in [12,13,14]. The point intensity decreases due to changes in the reflective properties of surfaces under adverse weather conditions. Figure 3 shows the point-intensity histogram for snow, rain, and fog using the real-world point cloud data shown in Figure 1. These altered point intensities might affect the accuracy of point cloud filtering, object detection, and segmentation algorithms that rely on intensity values, particularly deep-learning methods. We study the impact of point intensity on object detection in Section 5.

### 3.1. Noise Pattern

**Figure 4:**
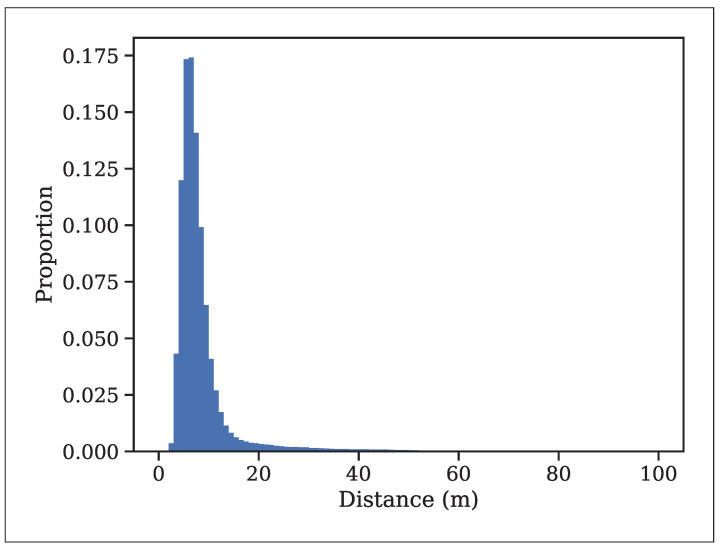
Snow noise distribution (%) vs. the distance from LiDAR averaged over the scans in the WADS dataset [8].

The type and intensity of adverse weather conditions influence the noise characteristics in LiDAR scans. Generally, the noise is scattered randomly in rain and snow and diminishes with increasing distance from the LiDAR sensor, as noted in [12]. Figure 4 illustrates the normalized histogram of snow points with respect to the distance from the LiDAR, averaged over all scans in the WADS dataset. In contrast, most of the noise observed in dense fog, dust, or rain mist often forms a “wall” where points are concentrated at the boundary of the fog or dust cloud. This wall-like noise pattern differs significantly from the scattered noise caused by rain and snow (Figure 1), presenting unique challenges for LiDAR-based perception systems. In [1], heavy rain is shown to have a fog-like effect on point clouds, with noise concentrated near the spray nozzle with dense misting of water. However, raindrops are randomly distributed, resulting in a noise pattern similar to that observed in snow. According to [15], light, moderate, or heavy rainfall does not substantially impact the LiDAR sensor’s performance.

### 3.2. Weather Intensity

The severity of adverse weather further influences the amount of noise in LiDAR scans. In heavy snow or dense fog, the number of noise points increases with weather intensity—more intense weather results in more noise. The effect is more pronounced in dense fog, where noise appears as a solid wall near the LiDAR sensor, reducing the number of feature points. This wall-like noise can occur where dust or mist accumulates. The noise pattern in dense fog severely impacts LiDAR’s usability for object detection to a certain distance, as shown in [14] for foggy weather in a controlled environment.

## 4. Point Cloud Filtering in Adverse Weather

### 4.1. Setup

In this section, we comprehensively evaluate several filtering methods for denoising point clouds in adverse weather conditions. The focus is on assessing the accuracy of noise removal and the processing time of these algorithms for filtering out noise caused by adverse weather.

#### 4.1.1. Filtering Algorithms

Several statistics-, intensity-, and deep-learning-based methods are available for removing adverse weather noise from point clouds. The statistics- and intensity-based filtering methods, while generally adaptable to different point clouds, require parameter tuning specific to LiDAR sensors and adverse weather for effective filtering. Deep-learning methods, such as those described in [16,17], learn point cloud features for noise filtering but require extensive and diverse training datasets to train a generalized model.

Statistics-based Filter: The statistics-based filter evaluated in this work includes radius outlier removal (ROR), statistical outlier removal (SOR), dynamic radius outlier removal (DROR) [18], and dynamic statistical outlier removal (DSOR) [8]. In ROR, a point is an inlier if the number of neighbors within a fixed radius exceeds a certain threshold. DROR adapts this approach by calculating the neighbor search radius for each point individually using the vertical LiDAR resolution. SOR determines a distance threshold using the mean and variance of the mean neighbor distance for all points in the point cloud and considers a point an inlier if its mean neighbor distance is below this threshold. DSOR refines this by dynamically calculating thresholds for each point based on its range, resulting in smaller distance thresholds for points closer to the LiDAR and larger thresholds for distant points.

Intensity-based Filter: Several intensity-based filter methods have been proposed for removing snow points, which typically have lower intensity values. Methods such as low-intensity outlier removal (LIOR) and dynamic distance-intensity outlier removal (DDIOR) use a two-step filtering process. In the first step, potential snow points (outliers) are identified using an intensity threshold, which may also include points of interest (e.g., surface points from objects). Hence, in the second step, a statistics-based method is used to re-classify points of interest as inliers. LIOR employs a ROR filter in the second step, while DDIOR uses custom dynamic thresholds that depend on point intensity and point range. LIOR was designed for Ouster LiDARs, which have a broad signal range (0 to 5500), whereas the WADS dataset used in this study has a small intensity range (0 to 255). Hence, we changed the LIOR intensity threshold from 0.167 to 1 in our experiments.

Deep-learning-based Filter: In this work, we evaluated 4DenoiseNet [16] deep-learning based denoising method. We used the 4DeNoiseNet model because it is trained on the SnowKitti dataset, which has the same LiDAR point cloud resolution as the WADS dataset, and because its pre-trained weights are available. 4DeNoiseNet utilizes spatial and temporal information through a novel k-nearest neighbors search convolution applied to consecutive point clouds.

#### 4.1.2. Dataset

**Figure 5:**
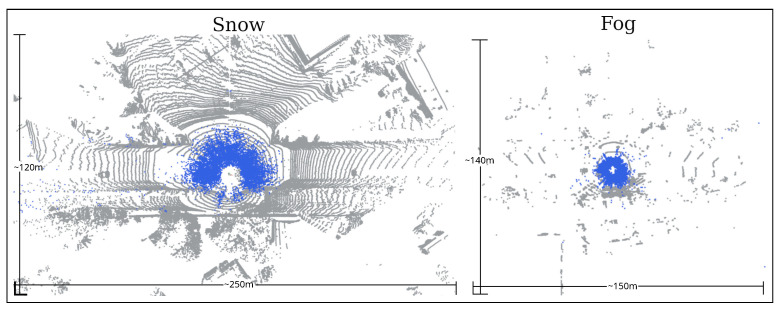
A top-view of point cloud from WADS Dataset [8] (snow) and Boreas Dataset (simulated fog). Blue color points represent the falling snow and simulated fog.

As mentioned in Section 2, we used the WADS dataset [8] for snowy weather and synthetically generated dense fog in the “boreas-objects-v1” sequence of Boreas dataset to evaluate filtering methods. Before filtering, we ensured point uniqueness by removing duplicate points with identical coordinates in the WADS dataset. Figure 5 illustrates a sample point cloud from the WADS dataset and synthetically generated dense fog in the Boreas dataset. WADS and Boreas datasets are collected using 64- and 128-channel resolution LiDARs, respectively. Point cloud resolution is downsampled to 64-channels for the Boreas dataset.

#### 4.1.3. Experiment Setup

All statistical- and intensity-based filtering methods were reimplemented in Python v3.10 using the SciPy and NumPy libraries to improve execution time and ensure a consistent setup for comparison. This re-implementation speeds up filtering compared to previous implementations of these methods by using the kDTree method from the SciPy spatial library, which parallelizes the neighbor search across four threads. The filtering was conducted on an Intel CPU with 32 GB of RAM (Intel Corporation, Santa Clara, CA, USA) and an Nvidia RTX 4090 (for the 4DeNoiseNet model) (Nvidia Corporation, Santa Clara, CA, USA). We used the default configuration of 4DeNoiseNet, with the model weights provided in the original repository. Table 2 lists the parameters used in point cloud filtering.

#### 4.1.4. Evaluation Metrics

The performance of each filtering algorithm was evaluated using precision, recall, and execution time metrics. Precision quantifies the algorithm’s accuracy in identifying and removing noisy points caused by adverse weather while preserving actual object points. Higher precision indicates more effective noise removal. Recall measures the algorithm’s ability to detect and eliminate all noisy points in the point cloud—higher recall indicates more effective filtering of noise. Execution time is also analyzed to identify algorithms suitable for real-time applications with processing speed requirements. Furthermore, we assessed the algorithms’ effectiveness in correctly classifying object points (especially vehicles and pedestrians) without incorrectly labeling them as outliers.

### 4.2. Filtering Algorithm Results and Discussion

Table 3 presents the results of point cloud filtering in snow and dense fog weather using various point cloud filtering algorithms. The results indicate that dynamic filtering methods (DROR, DSOR, and DDIOR) achieved higher precision and recall than other methods. Notably, due to their adaptability, dynamic statistics-based filters (DROR and DSOR) outperformed intensity-based filters in terms of object point misclassification rates, providing reliable performance across varying conditions.

Dynamic point cloud filters (DROR, DSOR, and DDIOR) consistently outperform other filtering methods on precision and recall metrics, achieving accuracy greater than 90% in both snowy weather and dense fog. The key factor contributing to the improved performance of dynamic filters is dynamic thresholding, which is a function of point distance from the sensor. This is more relevant for filtering a single scan from rotating LiDARS, which emit multiple light beams in a rotating or sweeping pattern to capture 3D surroundings. As the distance from the sensor increases, the spacing between consecutive beams increases, resulting in sparser point coverage at longer ranges. Dynamic filtering methods adapt to varying point distributions and preserve structural integrity. On the other hand, ROR, SOR, and LIOR are the worst-performing filtering algorithms, especially in dense fog, where most noise is concentrated near the LiDAR and fails to meet outlier criteria. Intensity-based filters may work better for LiDAR sensors with a wide range of point intensities.

Statistics-based filtering methods filter the distant points, reducing the effective LiDAR range. For ROR and SOR, the LiDAR point range decreased by 60–70% in snow and 80% in dense fog. However, dynamic statistics-based filters mitigate this limitation by dynamically calculating a threshold for each point, reducing LiDAR range by approximately 3% in snow and 60% in dense fog. A reason for the drastic range reduction in dense fog is a very sparse point cloud at a distance from the LiDAR. In contrast, intensity-based filters exhibited minimal range reduction (<1%) because filtering is applied within a certain distance from LiDAR. DDIOR, for example, considers points outside a 90 m radius as inliers in snowy weather. Since adverse weather introduces noise primarily near the LiDAR, filter algorithms should be applied only within a specified range. The reduction in point range may impact SLAM and localization algorithms, as discussed in Section 7.

**Figure 6:**
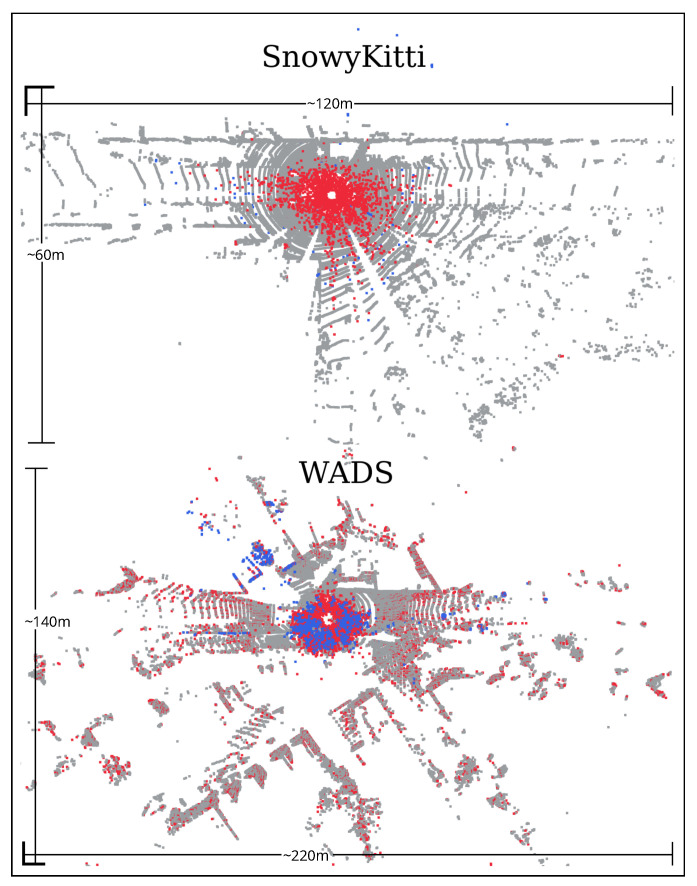
Result of 4DeNoiseNet point cloud filtering on SnowyKitti and WADS point clouds. Blue color points represent the noise due to snowfall and red color represent the detected outliers.

We evaluate the deep learning filtering method, 4DeNoiseNet, only under snowy weather conditions, as the model is trained to filter snow. The overall performance is worse than that of dynamic filters. The main reason is the difference in the training data (SnowyKitti) and test data (WADS) regarding the amount of noise/snowfall intensity. Figure 6 shows the noise and the detected outliers in the train (SnowyKitti) and test (WADS) datasets.

Regarding execution time, Table 3 shows that 4DeNoiseNet is the fastest filtering algorithm, with an average execution time of 8.5 ms. LIOR is the fastest in analytical filtering algorithms, with an average filtering time of 30 ms in snowy weather and 43.5 ms in dense fog. The reason for the increased execution time in dense fog for LIOR is that more points are concentrated near the LiDAR as noise, resulting in a longer execution time at the second filtering stage. The other filtering algorithms demonstrated comparable execution times of approximately 50 ms after incorporating parallelized neighbor search using Scipy’s kDTree implementation. We reported filtering execution times for a 64-channel LiDAR resolution (approximately 117,000 points); with higher resolutions, execution times might increase. Another critical comparison involves the algorithms’ ability to avoid classifying object points (e.g., vehicles, pedestrians, and cycles) as outliers, which is essential for object detection tasks. Dynamic filtering methods outperformed other filters, enhancing 3D object detection, as demonstrated in Section 5.

From the results, we can see that dynamic filtering methods offer promising results; however, they need to be tuned based on sensor characteristics. In this study, parameters were optimized for a 64- and 128-channel LiDAR with approximately 45° vertical FoV, which is typical for automotive-grade sensors. These settings should be tuned for sensors with significantly different specifications, such as Ouster OS-0 with a 90° FoV or static LiDARs. The challenge of selecting a filtering algorithm and tuning its parameters depends heavily on the point cloud structure and requires a more comprehensive study for broad applicability.

Overall, dynamic filtering methods effectively filter adverse weather-induced noise for rotating LiDARs. However, the selection and fine-tuning of the filtering algorithm depend on specific environmental conditions, required accuracy, and computational resources. A deep learning-based filter trained on a large, diverse dataset encompassing different LiDAR resolutions, adverse weather conditions, and weather intensities might yield a generalized filtering method.

## 5. Object Detection in Adverse Weather

### 5.1. Setup

In this section, we evaluate 3D object detection performance under adverse weather conditions. We focused on detecting the “vehicle” and “person” classes and on the impact of various point cloud filtering algorithms on detection accuracy in adverse weather. We also investigated how object detection accuracy changes as the minimum detection range from the sensor increases, since noise from adverse weather decreases with increasing distance from the LiDAR. Additionally, we assessed the impact of point intensity on object detection accuracy, which is generally reduced under adverse weather conditions. By conducting this comprehensive evaluation, we aimed to provide valuable insights into 3D object detection under adverse weather conditions and into how point cloud filtering algorithms affect object detection accuracy.

#### 5.1.1. Object Detection Model

We used the PointPillar 3D object detection model with a feature pyramid network (FPN) architecture [19], trained on the NuScenes dataset [20], for object detection in adverse weather conditions. The pretrained weights and model architecture were sourced from OpenMMLab’s MMDetection3D Library [21]. The PointPillar model is selected as it is independent of LiDAR resolution. The architecture comprises three main modules: a pillar feature net, a 2D CNN feature extractor backbone, and a detection head. The point cloud is converted into a pseudo-image by dividing it into 2D grids of fixed size in the XY-plane parallel to the ground; a feature vector is extracted for each grid from the points within it, forming the channel dimension of the pseudo-image. This pseudo image is then passed through the 2D CNN backbone to extract features for the detection head. The pseudo-image is in a bird’s-eye-view (BEV) perspective. The model weights trained on the NuScenes dataset were chosen for evaluation, as the point cloud characteristics (e.g., LiDAR resolution and point intensity range) and object categories in the NuScenes dataset are similar to those in the CADC [9] dataset used in this study.

#### 5.1.2. Dataset

For this evaluation, we utilized the CADC dataset [9] with snow weather conditions and the Boreas dataset with synthetically generated dense fog. The point cloud data in the CADC and Boreas datasets have 32- and 128-channel resolutions, respectively, and point intensities range from 0 to 255. The point cloud in the Boreas dataset is downsampled to a 32-channel resolution in this evaluation which refers to reducing the number of LiDAR scan lines (vertical channels) by selecting a subset of beams from the original sensor data, preserving the angular structure but lowering vertical resolution specific for rotating LiDARs like Ouster or Velodyne. We focused on detecting “vehicle” and “person” classes. Hence, the object class names in NuScenes, CADC, and Boreas datasets were re-labeled as per Table 4. Given the 50-m detection range of the pre-trained object detection model, we sorted the ground truth accordingly. Additionally, we filtered out ground truth and predicted labels when the number of points inside the bounding box was less than ten.

#### 5.1.3. Experiment Setup

We evaluated object detection using noisy and filtered point clouds. The filtering algorithms evaluated are ROR, SOR, DROR, DSOR, LIOR, and DDIOR with the parameters given in Table 5. The parameters differ from those in Table 2 because the LiDAR resolution is 32 channels instead of 64, which is compensated by increasing the search radius or the multipliers. Object detection was performed on an Nvidia RTX 4090 GPU.

#### 5.1.4. Evaluation Metrics

We reported the mean average precision (mAP) of 3D object detection using the BEV evaluation metric from the NuScenes dataset. Additionally, we assessed object detection performance at varying distances from the sensor—specifically within the ranges of 0–50 m, 3–50 m, 5–50 m, 10–50 m, and 20–50 m—to identify the effective distance at which detection remains reliable, even under adverse weather conditions. This analysis is crucial, as noisy points due to adverse weather tend to concentrate near the LiDAR sensor and manifest as noise in the 2D-BEV pseudo-image used for object detection with the PointPillar model.

### 5.2. Object Detection Results and Discussion

**Figure 7:**
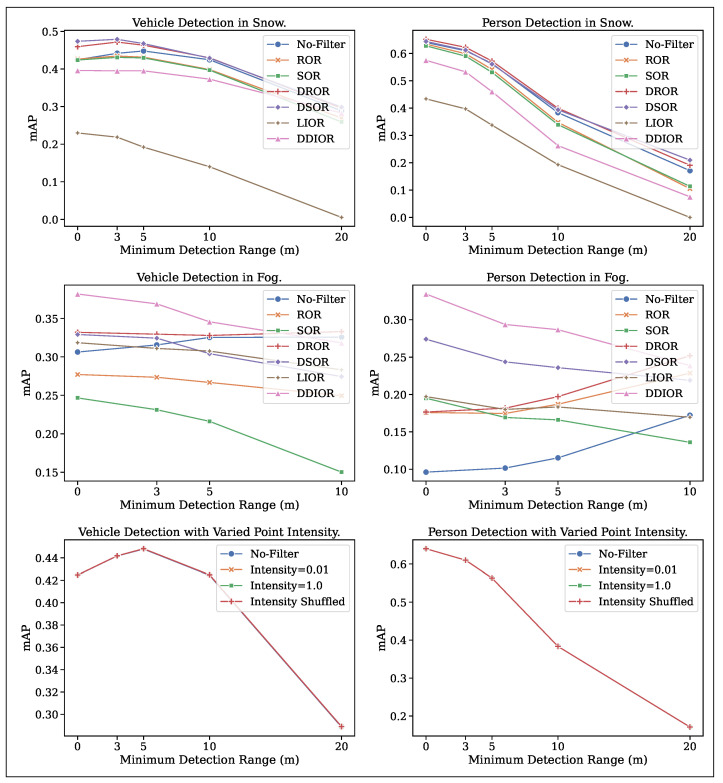
Mean average precision (mAP) of object detection in adverse weather conditions with detection ranges: 0–50 m, 3–50 m, 5–50 m, 10–50 m, and 20–50 m. The top and middle rows show the plot of object detection mAP in snowy and dense fog weather, and the bottom row shows the plot of the effect of point intensity on object detection mAP. The lines almost overlap in the bottom plots showing point intensity have negligible impact on 3D object detection. The improvement in object detection due to filtering is limited to a certain distance (∼10 m) from the LiDAR sensor for snowy weather.

Figure 7 illustrates the mAP plots for the “vehicle” and “person” classes, comparing detection accuracy using noisy and filtered point clouds and the effect of point intensity across different detection ranges. Point cloud filtering significantly enhances object detection in adverse weather, particularly for objects near the LiDAR sensor. This improvement, as indicated by the mAP of the “vehicle” class, demonstrates the effectiveness of dynamic filtering algorithms on object detection in adverse weather conditions. The improvement for the “person” class is less significant, as the CADC and Boreas datasets were collected while driving, and the pedestrians are generally far from the driving vehicle. Only 10.94% of pedestrians in the CADC dataset are within a 10m radius from the sensor.

**Figure 8:**
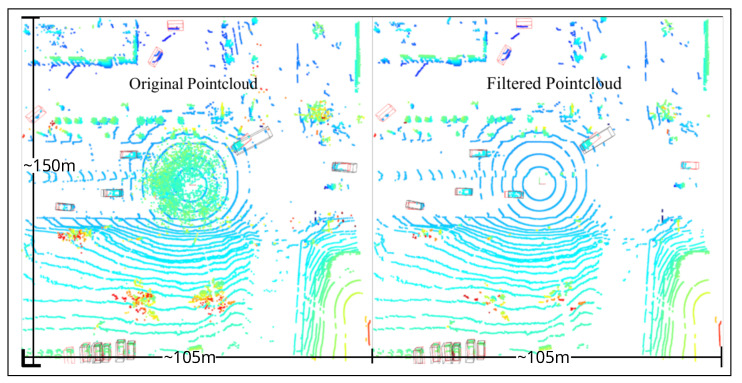
Object detection in a noisy and filtered point cloud (Ground Truth: Red boxes, Prediction: Black Boxes) from CADC dataset.

The results also highlight that the improvement in object detection due to filtering is limited to a certain distance from the LiDAR sensor, approximately 10 m in snowy weather and dense fog. The main reason for this in snow and foggy weather is due to noise concentration near the sensor, as discussed in Section 3. This finding is particularly relevant for 3D object detection algorithms that use BEV projection. Figure 8 shows the noisy and filtered point cloud in a BEV perspective, showing the difficulty in distinguishing the noise from the vehicle close to the LiDAR. However, the results might differ for 3D object detection algorithms that do not use BEV projection.

Only dynamic filtering algorithms improved object detection in adverse weather. Specifically, DSOR and DROR achieved higher detection accuracy (>0.45 mAP) in snowy weather, while DDIOR and DSOR performed better (>0.325 mAP) in dense fog. These results are confirmed by filtering the data presented in Table 3, which shows that dynamic filters yield the lowest percentage of object points detected as outliers. Object detection in snowy weather is better than in dense fog due to lower noise levels.

Detection plots also exhibited consistent trends regarding detection range, except for person detection in dense fog, where mAP improved for no-filter, ROR, and DROR as the detection range increased. This oddity arises from the high noise concentration near the LiDAR sensor in dense fog, which complicates object detection without effective filtering. However, as the distance increases, the noise diminishes, leading to better object detection.

We further analyzed the impact of point intensity on object detection accuracy by conducting experiments on noisy point clouds. Specifically, we modified the normalized point intensity in three ways: (i) setting the intensity of all points to 0.01, (ii) setting the intensity of all points to 1.0, and (iii) randomly shuffling the original point intensities. The results indicate that point intensity has a minimal effect on object detection accuracy, suggesting that lower intensity values in adverse weather conditions do not significantly affect detection performance. This result indicates the number of points forming the object’s surface rather than point intensity. However, point intensity is valuable in detecting reflective surfaces, such as reflective vests and signs, making it useful for person detection in applications like construction sites, mining, or ports.

Overall, our study underscores the importance of point cloud filtering in improving 3D object detection in adverse weather conditions. Dynamic filtering methods performed best among the tested algorithms, but should be selected and fine-tuned for the LiDAR type and specific weather conditions.

## 6. Localization in Adverse Weather

### 6.1. Setup

In this section, we evaluate 3D localization performance under adverse weather conditions. The core concept involves creating a map of the environment in clear weather and performing localization in adverse weather conditions, including snow, rain, and fog. The experiments used only IMU and LiDAR data, without incorporating other sensors, and provided insights into the limitations of localization algorithms in adverse weather conditions.

#### 6.1.1. Dataset

In this experiment, we used the Boreas dataset [4], which includes data collected using various sensors, including a 128-channel LiDAR, radar, cameras, 6-axis IMU, and wheel odometry, in different weather conditions (sunny, cloudy, rainy, and snowy weather) and includes the ground truth LiDAR poses. We used the sequences “boreas-2021-01-26-11-22” (heavily snowing weather), “boreas-2021-04-08-12-44” (sunny weather), “boreas-2021-04-08-12-44” (synthetic dense fog), and “boreas-2021-04-29-15-55” (mild to moderate rainy weather) for localization experiments.

#### 6.1.2. Global Map Creation

**Figure 9:**
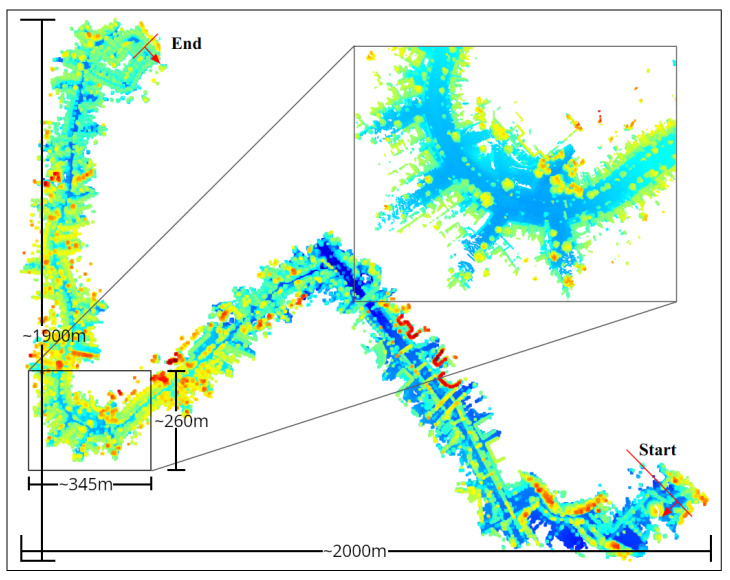
Map used for localization in adverse weather. The zoomed part shows a clearer map compared to a map generated without dynamic object filtering.

We used the “boreas-2020-12-18-13-44” sequence collected in sunny weather to create the map for localization. The map was generated using every 20th scan, and each scan was processed using a box filter with a range of (−75 m, 75 m) in the X and Y directions and (−3 m to 20 m) in the Z direction. Dynamic objects such as vehicles and persons were filtered out using the PointPillar network with FPN from OpenMMLab’s MMDetection3D library. Then, the DSOR point cloud filter is used for denoising, and the point cloud is downsampled using a voxel grid of size 0.1 m to reduce the number of points so the map can fit in memory. The processed scan was then transformed into the map frame using the ground-truth LiDAR pose and added to the map generated from previous scans. Figure 9 shows the localization map created using the “boreas-2020-12-18-13-44” sequence used for localization experiments.

#### 6.1.3. Localization Algorithm

The localization algorithm used in this study is LIORF-Localization (https://github.com/YJZLuckyBoy/liorf_localization, (accessed on 25 July 2024)), which is derived from the LIO-SAM SLAM algorithm [22]. The algorithm extracts surface-point features and then uses surface-to-map scan registration to align the current point cloud with the global map. Pre-processing includes a range filter to reduce the point cloud’s vertical resolution, grid-based downsampling, and rectifying LiDAR point distortion using IMU data.

#### 6.1.4. Evaluation Metrics

The absolute pose error (APE) metric was used to report localization accuracy in various weather scenarios. APE measures the overall accuracy of the estimated pose compared to the ground truth, providing insight into the algorithm’s long-term accuracy and drift over time. It assesses translation and rotation errors between the LiDAR’s estimated and ground-truth poses. EVO library [23] is used for the APE calculation. Before the APE calculation, the estimated and ground-truth trajectories were aligned using the Umeyama alignment.

#### 6.1.5. Experiment Setup

In each experiment, the algorithm was initialized using the correct initial pose. All other parameters of the localization algorithm were set to the default values provided in the library, except for the extrinsic transformation between the IMU and LiDAR sensor provided with the dataset. The vertical resolution of each scan is downsampled from 128 to 16 channels, and the points were filtered within a minimum and maximum range of 3 m to 100 m to speed up the algorithm. The experiments were conducted on an Intel Core i9 CPU with 16 cores running at 2.40 GHz and 32 GB of RAM (Intel Corporation, Santa Clara, CA, USA).

### 6.2. Localization Results and Discussion

Table 6 presents localization results across various weather conditions, showing different metrics for the translation part of the absolute pose error and its root mean square error (RMSE). The RMSE for localization in snowy weather was 11.7 cm, smaller than the errors in sunny and rainy weather. One reason for better localization in snowy weather is the slower vehicle speed, which results in more scans and a reduced mean of pose error. The average vehicle speed is 26.2 km/h, 35.4 km/h, and 34.2 km/h in snowy, sunny, and rainy weather while driving. The average vehicle speed was calculated when the vehicle was moving (speed ≥ 5 km/h) using the speed information provided in the dataset.

**Figure 10:**
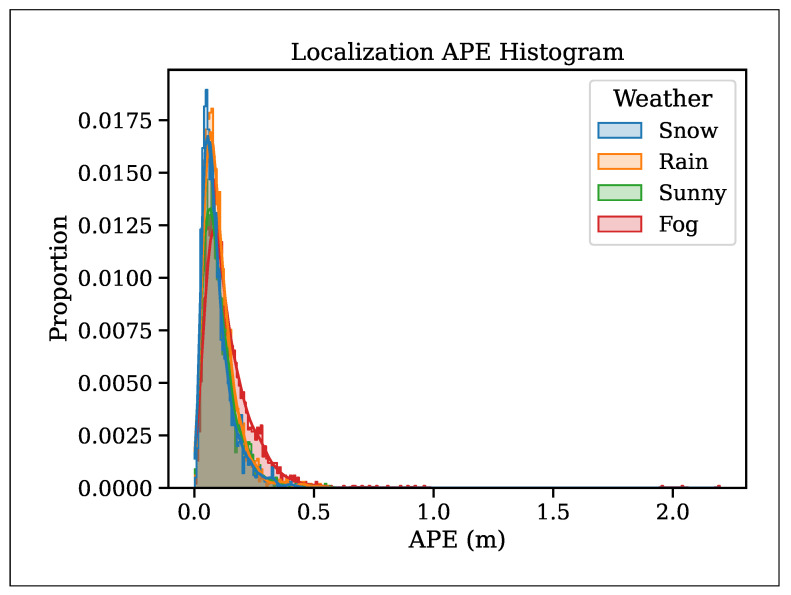
Histogram of localization absolute pose translation error (APE).

We plotted the normalized histogram of absolute translation errors shown in Figure 10 to reduce the influence of reduced vehicle speed on the analysis. The histogram reveals that the distribution of pose errors in snowy weather resembles that observed in sunny weather. This result demonstrates that the noisy points caused by snow did not significantly affect scan registration, as most of the noise was airborne and did not contribute to structural alignment in the map. This finding is consistent with the results reported in [24]. However, localization failed in dense fog; hence, we used filtered point clouds, which resulted in a translation RMSE of 0.182 m and a maximum translation error of 2 m. This result showed that dense fog adversely affects outdoor localization due to fewer feature points and a reduced point range. The localization might be affected in post-snowstorm conditions, where the map’s surface profile changes drastically and fewer features are available for registration.

The other parameter that affects the localization accuracy is the map resolution. In this work, we used a 0.1 m voxel size primarily to reduce memory consumption while maintaining sufficient geometric detail for reliable registration. The 0.1 m map resolution is dense and provides greater geometric detail for localization. However, in practical applications with real-time processing as a main concern, an ablation study of voxel size, scan resolution, and localization accuracy is needed.

Overall, adverse weather does not seem to affect the localization negatively. In heavy snow, the driving speed is less than in clear weather, which might result in improved performance compared to clear weather.

## 7. SLAM in Adverse Weather

### 7.1. Setup

This section outlines the experiments to evaluate the performance of 3D simultaneous localization and mapping (SLAM) algorithms in adverse weather conditions. As discussed in Section 3, adverse weather introduces noise into LiDAR data, which might impact the accuracy and reliability of the SLAM algorithms, particularly those relying solely on LiDAR. Our objective is to understand the extent of this impact and assess the effectiveness of various point cloud filtering algorithms in mitigating it. This study utilized only LiDAR and 6-axis IMU data to evaluate the performance of various SLAM algorithms in challenging weather conditions.

#### 7.1.1. SLAM Algorithm

The SLAM algorithm used in this evaluation is LIO-SAM [22], HDL-Graph SLAM [25], and DeepPointMap [26]. LIO-SAM and HDL-Graph SLAM are state-of-the-art methods known for their robustness, accuracy, and real-time performance, and DeepPointMap is a newly proposed deep-learning-based SLAM method.

LIO-SAM uses 9-axis IMU data (accelerometer, gyroscope, and orientation) to remove motion distortion from LiDAR data, perform point cloud filtering, and extract two feature point clouds: one containing corner points and the other containing surface points. The algorithm employs a combination of corner-to-corner and surface-to-surface feature registration techniques to estimate pose in dynamic environments accurately. Since the Boreas dataset contains only 6-axis IMU data (accelerometer and gyroscope), we used the identity orientation vector to make the dataset compatible with the LIO-SAM algorithm.

HDL-Graph SLAM has three main steps: prefiltering, scan matching, and creating and optimizing a pose graph with floor detection and loop closure constraints. The prefiltering step, similar to LIO-SAM, involves a range-based filter and downsampling of LiDAR resolution. Fast-GICP [27] is employed for scan matching for LiDAR odometry and loop closure detection. We also used HDL-Graph SLAM’s floor-detection method as an additional constraint on the pose graph. The estimated LiDAR odometry is corrected by optimizing the pose graph with loop-closure and floor-detection constraints. We did not use IMU data in HDL-Graph SLAM.

DeepPointMap is a LiDAR-only pose-graph SLAM approach that uses a deep-learning method to extract highly representative and sparse neural descriptors from point clouds, which are used with a decoder to estimate the pose between the pointclouds.

#### 7.1.2. Dataset

In this evaluation, we used the Boreas dataset [4], which includes point clouds and IMU data collected under three different weather conditions: sunny, snowy, and rainy weather. We used the same sequences “boreas-2021-01-26-11-22” (heavily snowing), “boreas-2021-04-08-12-44” (sunny weather), “boreas-2021-04-08-12-44” (synthetic dense fog), and “boreas-2021-04-29-15-55” (mild raining and overcast weather) used in localization experiments. Additionally, we used LiDAR-fog-sim [7] to simulate dense fog point cloud in “boreas-2021-04-08-12-44” sequence.

The dataset provides ground truth poses, enabling a detailed comparison of SLAM performance across these conditions. The sunny weather data serves as the baseline, representing optimal conditions with minimal noise. In contrast, the snowy, rainy, and foggy weather data introduce varying degrees of noise, allowing us to assess the impact of these adverse conditions on SLAM performance.

#### 7.1.3. Experiment Setup

In addition to evaluating the SLAM algorithm’s performance in different weather conditions, we also assessed the impact of various point cloud filtering algorithms on SLAM accuracy. Before running SLAM, we used SOR, DSOR, and DDIOR to filter the point clouds offline. The point cloud filtering parameters are the same as in Table 2. The other LIO-SAM parameters were set as the default values provided in the library, except for the extrinsic transformation between IMU and LiDAR. In the case of DeepPoinMap, we downsample the point cloud from 128-channels to 64-channels, since the model was trained on the KITTI dataset.

#### 7.1.4. Evaluation Metrics

We used two metrics to evaluate the SLAM algorithm’s performance: absolute pose error (APE) and relative pose error (RPE). APE measures the algorithm’s long-term accuracy and drift over time, while RPE assesses the accuracy of pose estimation over short intervals, highlighting the algorithm’s ability to maintain consistent localization and mapping in the presence of noise. We used the EVO library to calculate the various metrics. Before estimating APE and RPE, the ground-truth and estimated trajectories were aligned using the Umeyama alignment to ensure reliable metrics.

### 7.2. SLAM Results and Discussion

**Figure 11:**
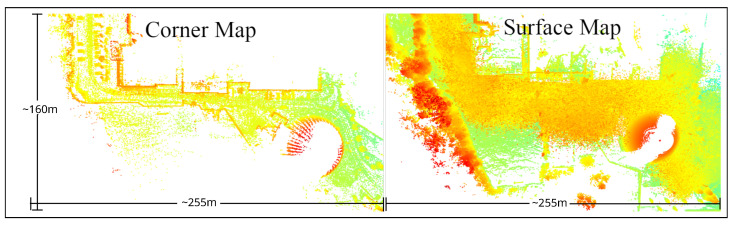
Part of the corner and surface feature maps from the Boreas dataset generated by the LIO-SAM SLAM algorithm in snowy weather.

Table 7 reports the translation RMSE of the APE and RPE metrics of various SLAM algorithms with and without point cloud filtering. The best SLAM result, i.e., the lowest translation APE, is 8.45 m, obtained in snowy weather using LIO-SAM without filtering. HDL-Graph SLAM worked but produced higher pose errors than filtered point clouds in snowy weather. As expected, DeepPointMap failed in snowy weather without point cloud filtering, as the model was not trained on heavily noisy data. All SLAM algorithms failed in dense fog due to extreme noise and reduced point range. The reason for the lower APE of LIO-SAM in snowy weather is two-fold: first is reduced speed in snowy conditions, resulting in smaller pose differences between consecutive scan timestamps, and second is better scan registration due to corner point features being less affected by falling snow, as shown in Figure 11.

**Figure 12:**
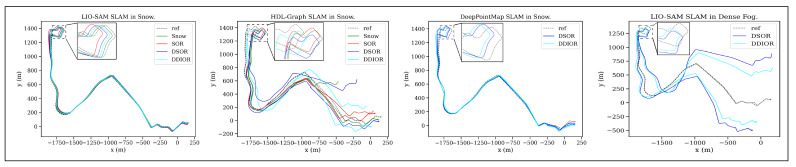
SLAM trajectory in snowy and foggy weather with and without point cloud filtering plotted after aligning the estimated trajectory with ground truth using Umeyama alignment.

When examining the effect of point cloud filters on SLAM in snowy weather and dense fog, we observed that dynamic filtering methods resulted in lower APE errors, especially for HDL-Graph SLAM and DeepPointMap. In dense fog, even point cloud filters were ineffective for SLAM, and only LIO-SAM did not fail drastically. The main reasons are the low number of point features for registration. These results underscore the importance of selecting an appropriate point cloud registration method. DeepPointMap’s comparable performance to LIO-SAM without fine-tuning demonstrates the effectiveness of deep-learning methods when using a point cloud with sufficient features and point range. Figure 12 shows the trajectory of successful runs for various SLAM algorithms with and without point cloud filtering.

While we did not observe a significant negative impact on SLAM despite the noise from heavy snowfall, it is important to note that the resulting maps may contain considerable noise, potentially affecting their usefulness. Dense fog, on the other hand, proved the most challenging condition for SLAM, leading to unsuccessful runs with various SLAM and point cloud filtering algorithms.

In practice, SLAM is often performed with a multi-sensor configuration that may include wheel odometry, GPS, or radar, which can compensate for errors observed with LiDAR-only SLAM. Even considering only LiDAR, heavy snow did not negatively affect SLAM and even led to lower pose errors than in clear weather. However, creating a map under adverse conditions might not be preferable due to some noise in the point cloud, even after filtering.

## 8. Discussion

Adverse weather conditions, such as rain, snow, and fog, degrade LiDAR point cloud quality, introducing noisy points, reducing the effective point range, and lowering point intensity. Noise points from snowflakes, raindrops, and fog particles are transient and scattered throughout the point cloud, resulting in substantial noise, particularly under severe weather conditions. This noise can lead to incorrect object detection or point cloud alignment, lowering localization and SLAM accuracy. Additionally, reduced point intensity in adverse weather may compromise the accuracy of filtering, object detection, and segmentation algorithms, especially those using deep learning.

Point cloud filtering might be an effective solution for removing noise caused by adverse weather, resulting in several filtering algorithms, such as dynamic statistical outlier removal (e.g., DROR and DSOR) and intensity-based outlier removal methods (e.g., LIOR and DDIOR). Our evaluation shows that dynamic filtering methods are highly effective at reducing noise and enhancing 3D object detection accuracy. However, these filtering methods reduce the overall range of the point cloud, affecting localization or SLAM tasks negatively. In contrast, intensity-based filtering methods preserve the point range but may misclassify object points as noise, resulting in lower object detection accuracy, as shown in Figure 7. These filtering techniques are designed for specific weather conditions and LiDAR sensor specifications, requiring parameter fine-tuning for each sensor, weather type, and perception task.

We analyzed the impact of adverse weather noise and the effectiveness of point cloud filtering methods on downstream perception tasks, object detection, localization, and SLAM. Our findings indicate that adverse weather reduces object detection accuracy within a specific range around the LiDAR, but point cloud filtering improves it. We also investigated the influence of point intensity on object detection performance by setting point intensity values to constants (0.01 and 1.0) and by randomly shuffling point intensities, but found a minimal impact on performance. This result suggests that the spatial relationship between points is more critical than point intensity values for object detection. However, point intensity is essential for detecting reflective objects such as reflective vests or traffic signs, making it more relevant in off-road applications like construction sites and mines.

Contrary to the results of object detection in adverse weather, we observed a minimal impact of heavy snowfall on localization and SLAM performance when using only LiDAR sensors; however, dense fog was more challenging. Localization and SLAM using LIO-SAM algorithms performed better in adverse weather, possibly due to more consecutive scans resulting from lower vehicle speed and the availability of necessary features for point cloud alignment. However, LiDAR-only localization algorithms are constrained by reduced point range and insufficient features (especially in dense fog in outdoor scenarios) for aligning the point cloud with the map. LiDAR-only SLAM algorithms face similar challenges. This study primarily focused on LiDAR perception; integrating additional sensors, such as wheel odometry, GPS, and radar, could enhance localization and SLAM accuracy under adverse weather conditions. Such integration has the potential to improve localization and SLAM performance, especially the odometry information using radar sensors [28], which are generally unaffected by adverse weather and will be investigated in the future.

In addition to noise, adverse weather introduces other challenges to LiDAR perception which are not explicitly investigated in the paper. For instance, snow accumulation on the ground or on objects after heavy snowfall can alter surface profiles, creating misleading features and causing localization errors and incorrect object detection. It also complicates lane recognition using LiDAR or camera sensors. Reduced LiDAR range limits long-range object detection, and dense fog severely impacts localization and SLAM in outdoor environments. Other research has explored how increasing the LiDAR’s operating wavelength from 905 nm to 1550 nm could improve performance under adverse weather conditions [29,30].

To the best of the author’s knowledge, no current advanced driver-assistance systems (ADAS) or autonomous vehicle systems actively detect weather conditions and integrate this information into their processing pipeline as explored in [31]. Leveraging weather information (both type and intensity) could enhance system robustness by guiding filtering algorithms and incorporating the limitations of LiDAR and associated algorithms. While some research has explored weather detection using cameras [32,33] and LiDAR-based sensors [14], these algorithms generally do not estimate weather intensity, which could be valuable for optimizing system performance.

The insights gained from this study advance our understanding of LiDAR perception in adverse weather conditions. We have highlighted the limitations and challenges posed by environmental factors and proposed practical solutions to mitigate their impact, ultimately aiming to improve the safety and reliability of autonomous driving in real-world, adverse weather conditions.

## 9. Conclusions

In this study, we examine the impact of adverse weather on LiDAR perception algorithms for autonomous driving, analyzing how these conditions degrade point cloud data and evaluating methods to mitigate their effects. Our results show that point cloud filtering techniques are highly effective at reducing noise caused by adverse weather, thereby enhancing the performance of object detection algorithms. In contrast, localization and SLAM tasks appear somewhat resilient to weather-related noise. Still, incorporating real-time weather information into the autonomous driving pipeline is promising. This integration could enhance the reliability and safety of LiDAR perception systems by providing valuable insights into the limitations posed by different weather conditions, ultimately enabling the development of more robust autonomous vehicles capable of navigating safely across diverse environmental conditions. 

## Figures and Tables

**Table 1:** Dataset used in this work for evaluation of LiDAR perception algorithms in adverse weather. We used synthetic (syn) foggy condition generated using LiDAR-fog-sim [7].

Weather	Task	Dataset	Sequence	Line Count	Scene
Snow	Filtering	WADS [8]	-	64 ch	Urban/Sub-urban
	Object Detection	CADC [9]	-	32 ch	Urban/Sub-urban
	Localization	Boreas [4]	boreas-2021-01-26-11-22	128 ch	Urban/Sub-urban/Highway
	SLAM	Boreas	boreas-2021-01-26-11-22	128 ch	
Rain	Localization	Boreas	boreas-2021-04-29-15-55	128 ch	
	SLAM	Boreas	boreas-2021-04-29-15-55	128 ch	
Fog	Filtering	Boreas (syn)	boreas-objects-v1	128 ch	Urban/Sub-urban
	Object Detection	Boreas (syn)	boreas-objects-v1	128 ch	
	Localization	Boreas (syn)	boreas-2021-04-08-12-44	128 ch	
	SLAM	Boreas (syn)	boreas-2021-04-08-12-44	128 ch	

**Table 2:** Point cloud filtering algorithm parameters used in the evaluation of snow and fog point filtering.

Method	Parameters	Snow	Fog
ROR	Min. Neighbors	3	
	Search Radius	0.2 m	
SOR	Num. Neighbors	3	
	Standard Deviation Multiplier	0.01	0.015
DROR	Min. Neighbors	2	
	Radius Multiplier	2	
	Azimuth Angle	0.625	
DSOR	Num. Neighbors	3	
	Range Multiplier	0.05	
	Standard Deviation Multiplier	0.05	
LIOR	Detection Range	71.2	30
	Point Intensity Threshold	1.0	
	Search Radius	0.1 m	0.15 m
	Min. Neighbors	3	
DDIOR	Num. Neighbor	3	

**Table 3:** The result of point cloud filtering averaged over all the LiDAR scans in the WADS Dataset (snowy weather) and Bores Dataset (synthetical dense fog). An upward arrow (↑) indicates that higher values are better, and a downward arrow (↓) indicates that lower values are better. Bold values indicate the best result achieved for each metric under each weather condition.

	Filter	Accuracy ↑	Precision ↑	Recall ↑	F1 Score ↑	Object as Outlier (%) ↓	Exec Time (ms) ↓	Max Range Reduction (%) ↓
Snow	ROR	0.717	0.182	0.287	0.210	13.496	46.864	63.480
	SOR	0.673	0.186	0.411	0.241	22.231	49.927	73.823
	DROR	0.929	**0.813**	0.658	0.715	**1.185**	50.598	2.782
	DSOR	0.911	0.608	0.890	0.706	6.167	51.575	3.169
	LIOR	0.900	0.600	0.803	0.675	15.344	30.018	**0.000**
	DDIOR	**0.935**	0.693	**0.903**	**0.776**	13.905	52.911	0.461
	4DenoiseNet	0.820	0.353	0.321	0.315	13.218	**8.464**	0.170
Fog	ROR	0.409	0.595	0.131	0.214	14.928	44.536	78.286
	SOR	0.434	0.651	0.181	0.282	17.294	48.751	80.521
	DROR	0.647	0.950	0.458	0.616	**2.910**	49.155	59.343
	DSOR	**0.933**	0.918	**0.977**	**0.946**	15.199	54.959	59.905
	LIOR	0.467	0.729	0.220	0.336	14.249	**43.495**	**0.000**
	DDIOR	0.918	**0.953**	0.911	0.931	16.914	55.058	58.657

**Table 4:** Re-labeling of the Nuscene and the CADC object labels for object detection experiments.

	CADC	NuScene	Boreas
vehicle	Car, Truck, Bus	car, truck, trailer, bus, construction vehicle	Car, Misc
person	Pedestrian, Pedestrian With Object	pedestrian	Pedestrian

**Table 5:** Point cloud filtering algorithm parameters used in the evaluation of object detection in adverse weather.

Method	Parameters	Snow	Fog
ROR	Min. Neighbors	3	
	Search Radius	0.4 m	
SOR	Num. Neighbors	3	
	Standard Deviation Multiplier	0.03	
DROR	Min. Neighbors	3	
	Radius Multiplier	2	
	Azimuth Angle	1.25	
DSOR	Num. Neighbors	3	
	Range Multiplier	0.05	
	Standard Deviation Multiplier	0.1	
LIOR	Detection Range	71.2	30
	Point Intensity Threshold	1.0	
	Search Radius	0.2 m	0.3 m
	Min. Neighbors	3	
DDIOR	Num. Neighbor	3	

**Table 6:** Localization absolute translation error metrics in various weather scenarios. In case of dense fog, the localization failed without pointcloud filtering; hence, results with DSOR filtering are reported in dense fog. A downward arrow (↓) indicates that lower values are better. Bold values indicate the best result achieved for each metric.

	RMSE (m) ↓	Mean (m) ↓	Max (m) ↓	Min (m) ↓
Snow	**0.117**	**0.096**	**0.494**	0.005
Sunny	0.129	0.106	0.563	0.005
Rain	0.126	0.104	0.562	**0.002**
Fog-DSOR	0.182	0.140	2.196	0.005

**Table 7:** SLAM absolute pose error (APE) and relative pose error (RPE) metrics for various weather and point cloud filtering algorithms. ‘F’ represents the SLAM failure. Bold values indicate the best result from each SLAM algorithm.

		APE RMSE (m)			RPE RMSE (m)		
	Filter	HDL SLAM	LIO SAM	DeepPointMap	HDL SLAM	LIO SAM	DeepPointMap
Sunny	-	53.148	17.416	19.846	14.905	0.195	**0.213**
Snow	-	188.748	**8.504**	F	22.474	0.115	F
	SOR	68.306	27.163	F	4.882	**0.063**	F
	DSOR	38.552	18.909	**18.480**	**3.018**	0.066	0.299
	DDIOR	141.290	13.735	29.520	9.521	0.079	0.240
Rain	-	**11.735**	25.852	40.062	5.436	0.191	0.226
Fog	-	F	F	F	F	F	F
	SOR	F	F	F	F	F	F
	DSOR	F	379.029	F	F	0.088	F
	DDIOR	F	289.260	F	F	0.079	F

## Data Availability

The data used for experiments in this article is publicly available and has been cited. The self-collected data used for noise analysis is available in the Github repository https://github.com/hgupta01/LiDAR-in-Adverse-Weather-Evaluation (accessed on 24 November 2025).
